# Supplementary material for: Size-Dependent Impact of Magnetic Nanoparticles on Growth and Sporulation of Aspergillus niger
Source: Molecules. 2022 Sep 9;27(18):5840. doi: 10.3390/molecules27185840 (PMC9502663; doi:10.3390/molecules27185840)
Supplement: Supplementary file 1 [file molecules-27-05840-s001.zip › molecules-1897740-supplementary.pdf]

Size-dependent impact of magnetic nanoparticles on growth and sporulation of *Aspergillus niger*

Zhishang Shi [1], Yan Zhao [1], Shuo Liu [1,2] Yanting Wang [1], and Qilin Yu [1,*]

[1] Key Laboratory of Molecular Microbiology and Technology, Ministry of Education, Department of Microbiology, College of Life Sciences, Nankai University, Tianjin 300071, China

[2] Tianjin Key Laboratory of Environmental Remediation and Pollution Control, College of Environmental Science and Engineering, Nankai University, Tianjin 300350, China

* Correspondence: yuqilin@mail.nankai.edu.cn

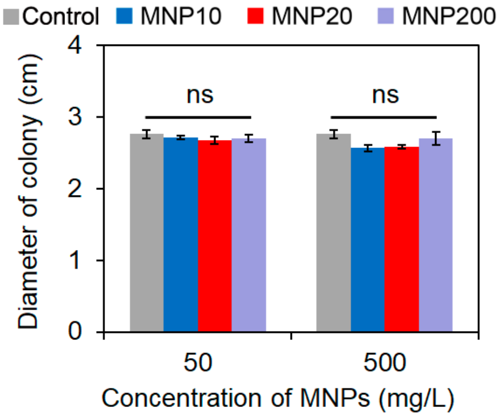

Figure S1. Effect of the MNPs on colony growth of *A. niger* on the PDA plates.
